# Flower-like ZnO Nanostructures Local Surface Morphology and Chemistry

**DOI:** 10.3390/nano12152666

**Published:** 2022-08-03

**Authors:** Monika Kwoka, Elisabetta Comini, Dario Zappa, Jacek Szuber

**Affiliations:** 1 Department of Cybernetics, Nanotechnology and Data Processing, Faculty of Automatic Control, Electronics and Computer Science, Silesian University of Technology, 44-100 Gliwice, Poland; jacek.szuber@polsl.pl; 2 SENSOR Laboratory, Department of Information Engineering, Brescia University, 25123 Brescia, Italy; elisabetta.comini@unibs.it (E.C.); dario.zappa@unibs.it (D.Z.)

**Keywords:** ZnO nanoflowers, surface morphology, surface chemistry, surface reactivity, thermal desorption

## Abstract

This work presents the results of comparative studies using complementary methods, such as scanning electron microscopy (SEM), X-ray photoemission spectroscopy (XPS), and thermal desorption spectroscopy (TDS) to investigate the local surface morphology and chemistry of flower-like ZnO nanostructures synthesized by the thermal oxidation technique on native Si/SiO_2_ substrates. SEM studies showed that our flower-like ZnO nanostructures contained mostly isolated and irregular morphological low-dimensional forms, seen as rolled-up floss flowers, together with local, elongated, complex stalks similar to Liatris flowers, which contained joined short flosses in the form of nanodendrites. Beyond this, XPS studies showed that these nanostructures exhibited a slight surface nonstoichiometry, mostly related to the existence of oxygen-deficient regions, combined with strong undesired C surface contamination. In addition, the TDS studies showed that these undesired surface contaminations (including mainly C species and hydroxyl groups) are only slightly removed from the surface of our flower-like ZnO nanostructures, causing an expected modification of their nonstoichiometry. All of these effects are of great importance when using our flower-like ZnO nanostructures in gas sensor devices for detecting oxidizing gases because surface contamination leads to an undesired barrier for toxic gas adsorption, and it can additionally be responsible for the uncontrolled sensor aging effect.

## 1. Introduction

Zinc oxide (ZnO) is an intrinsically n-type oxide semiconductor with a wurtzite structure that belongs to the group of transparent conductive oxides (TCOs). Owing to its unique optical properties (wide bandgap of 3.37 eV and large exciton binding energy of 60 meV), as well as peculiar electronic properties (high electronic mobility up to 2 cm^2^/V·s), in recent years, it has been used mainly in optoelectronics and photovoltaics [1,2,3].

Recently, ZnO has become one of the most investigated metal oxide (MOX) materials for potential applications in conductometric gas sensors for the detection of various active and toxic gases in many areas, mainly in relation to environmental monitoring [4,5]. MOXs are suited to this application because of their excellent sensing response and good selectivity, combined with thermal and chemical stability, as well as their relatively simple and low-cost fabrication [6,7,8].

In general, it is well-known that the gas sensing mechanism of ZnO, similar to other MOX materials, is mainly determined by the surface properties of its space charge layer (SCL), which are related to its surface/subsurface morphology. Moreover, it is a commonly accepted idea that a higher surface-to-volume ratio may enhance the performance of gas sensors with respect to coarse micrograined materials [9]. Moreover, for ZnO conductometric gas sensors, the electrical resistance is highly dependent on the presence of oxidizing and reducing gases. In the case of oxidizing gases, oxygen molecules coming from atmospheric air, after adsorption at the ZnO surface, cause the extraction of electrons from its conduction band, resulting in an increase in its resistance. In turn, in the case of reducing gases adsorbed at the ZnO surface, after interaction with oxygen ions, free electrons are donated to the conduction band, resulting in a decrease in the electrical resistance of the device [10]. The gas adsorption mechanism and sensing response of ZnO-based gas sensors are significantly affected by the gas concentration in combination with the working temperature [10].

After several years of intense research, it was demonstrated that the enhanced performance of ZnO gas sensors can be achieved relatively easily by using low-dimensional nanostructured forms. For ZnO, several fast-growth directions are available, allowing for the preparation of numerous ZnO nanostructures with various crystal planes and slightly different surface properties. This is a crucial point with respect to their potential gas sensor applications [11]. An overview of recent developments in the area of conductometric gas sensors based on various low-dimensional ZnO nanostructures can be found in two review papers [12,13]. Among the various low-dimensional ZnO nanostructures with their relative size- and morphology-dependent properties and gas sensor performance levels, in recent years, most effort has been devoted to 1D nanostructures, such as nanowires, nanorods, nanofibers, nanolines, nanobelts, nanoneedles, nanorings, and nanohelices. These peculiar morphologies were reviewed by Kumar et al. [11], Tonezzer et al. [14], Galstyan et al. [15], and Rackauskas et al. [16]. Furthermore, the size- and morphology-dependent properties and related gas sensor performance features of 2D ZnO nanostructures, such as nanosheets, nanowalls, nanoflakes, nanoplates, and nanodisks, were thoroughly reviewed by Tonezzer et al. [14] and Leonardi [17]. Moreover, in recent years, increasing attention has been devoted to transforming low-dimensional nano-sized building blocks into three-dimensional (3D) complex hierarchical structures. The main reason to do so is that 3D ZnO hierarchical nanostructures, with respect to the single-morphological nanostructures, usually inherit excellent properties related not only to the high surface-to-volume ratios but also to their suitability for potential applications in the bottom-up fabrication of functional devices including sensors [18].

Among the 3D ZnO hierarchical structures, the flower-like morphology is one of the most common forms, especially for potential conductometric gas sensor applications. In these 3D structures, the basic building blocks are mainly nanorods [19,20,21,22,23,24,25,26,27,28,29,30], nanosheets [28,29,30], nanopyramids [31], nanoplates [32], nanoflakes [33], and nanobundles [34]. In this context, flower-like ZnO nanostructures based on nanorods have been obtained primarily by the hydrothermal method [20,21,22,23,24,25,26], whereas 3D structures of nanosheets have been obtained mainly by wet chemical deposition [28,30]. Moreover, nanopyramids, nanoplates, and nanoflakes have been obtained primarily by the hydrothermal method [31,32] or the solvent version [33], whereas for nanobundles, the electrodeposition method was proposed [34]. Flower-like ZnO nanostructures have mainly been used to detect selected gases such as carbon monoxide (CO) [20,21,24,25,26,31] and nitrogen dioxide (NO_2_) [20,25,26,29,31,33], simple alcohols such as methanol (CH_3_OH) [21,22,27,28,29] and ethanol (C_2_H_5_OH) [20,21,22,24,26,27,28,32], acetone (C_3_H_6_O) [20,21,22,27,30], aromatic hydrocarbons such as benzene (C_6_H_6_) [26] and toluene (C_7_H_8_) [22], simple hydrocarbons such as methane (CH_4_) [25,27,31], ammonia (NH_3_) [25,27], hydrogen sulfide (H_2_S) [21,27], sulfur dioxide (SO_2_) [23], ozone (O_3_) [19], molecular hydrogen (H_2_) [21], and nitrogen oxide (NO) [33].

However, even after many years of exploring the fundamental gas sensor parameters of flower-like ZnO nanostructures, including their sensitivity and selectivity, as well as the response and recovery times, the reason for the undesired aging effect associated with their use has not been fully elucidated. This may be related to the fact that up to now, in characterizing these nanostructures, most research groups have focused primarily on determining their morphology by using a combination of electron microscopic methods such as scanning electron microscopy (SEM) [19,21,24,25,26,27,28,29,30,33,34] and transmission electron microscopy (TEM) [20,22,25,26,27,28] as well by determining their bulk crystallinity, phases, and chemistry by a combination of X-ray diffraction (XRD) [19,20,21,22,23,24,25,26,27,28,29,30,31,32,33,34] and optical spectroscopic methods such as Fourier-transform infrared spectroscopy (FTIR) [22,31,33], photoluminescence spectroscopy (PL) [20,21,22,24,31,33], Raman spectroscopy (RMS), and energy-dispersive X-ray spectroscopy (EDX) [23,27]. What is crucial is that most of the above-mentioned experimental techniques are bulk sensitive, whereas, as is well known, during gas interactions, the surface adsorption/chemisorption processes of gaseous species take place at the surface and within the subsurface region of gas sensor materials. These processes directly cause charge redistribution within the surface space-charge region of sensor material, related to the Debye length (LD), leading to the appearance of surface band bending effects, which play a crucial role in the specific surface conduction mechanism. This effect is also observed in the various nanoforms of ZnO, for which the carrier concentration reaches the value of ~10^18^ cm^−3^, and a surface depletion region with upward band bending is usually observed at a depth of a single nanometer [10,12,13]. Thus, it is evident that apart from the information concerning the local surface crystallinity and morphology, the local surface and subsurface chemistry of flower-like ZnO nanostructures, including their stoichiometry (combined with the undesired surface contaminations), should also be considered for the analysis and control of the gas sensing effect in various gas atmospheres. This is crucial for their potential application in the design and construction of novel gas sensor devices based on this material. Currently, one of the best methods for obtaining detailed surface chemical information is X-ray photoemission spectroscopy (XPS), which is similar to the above-mentioned surface space-charge region and comparable to the material LD. Recently, this method has been successfully used by our group to study the surface chemistry of low-dimensional SnO_2_ nanowires [35] and SnO_2_ nanolayers [36,37,38], and nanostructured ZnO porous, thin films [39]. Surprisingly, up to now, only limited studies are available in the literature reporting on the use of the XPS to characterize the surface chemistry and related effects of flower-like ZnO nanostructures [23,25,31,33,34]. To fill this research gap, in this work, we present the results of experimental studies on the specific local surface properties of flower-like ZnO nanostructures performed by using three comparative methods: SEM to observe the surface morphology, XPS to determine the local surface chemistry (nonstoichiometry with undesired surface contamination, specific surface bonding of basic atoms) and TDS for the additional control of surface adsorption/desorption effects of residual gases at the surface, as adsorbed from the residual atmosphere. The research activities performed in this work can be treated as a novel approach with respect to the available literature.

## 2. Materials and Methods

### 2.1. Preparation of Flower-like ZnO Nanostructures

Hierarchical flower-like ZnO nanostructures (NFs) were deposited on pristine Si(100)/SiO_2_ substrates at SENSOR Lab, Brescia University, Italy, using the following procedure. Si samples (10 × 10 mm^2^) were sonicated in acetone for 15 min, rinsed in distilled water, and then dried in pure air to remove dust and organic residues. The thermal oxidation technique was used to synthesize the nanostructures. A metallic zinc layer (thickness = 3 μm) was deposited by RF magnetron sputtering at room temperature using a 50 W argon plasma (pressure = 5.0 × 10^−3^ mbar). Finally, sputtered samples were placed in a tubular furnace and oxidized at 500 °C for 12 h in a pure-oxygen environment at atmospheric pressure, resulting in the formation of a homogeneous 3D porous, flower-like layer on the samples. A more precise description of the technological procedure was provided in our previous work [40].

### 2.2. Surface Characterization of Flower-like ZnO Nanostructures

For the complex characterization of the surface properties of hierarchical flower-like ZnO nanostructures, comparative experiments were performed using the SEM, XPS, and TDS techniques. The surface morphology was investigated using a high-resolution scanning electron microscopy (HR SEM) technique, as carried out at SENSOR Lab, Brescia University, Italy, using a field emission SEM (TESCAN MIRA3 Model, Brno, Czech Republic) operated at 10 kV. The surface chemistry of the flower-like ZnO nanostructures, including the stoichiometry and undesired surface contaminations, was determined by XPS before and after the TDS experiments. These combined XPS and TDS studies were performed at the Laboratory of Nanotechnology and Electronic Materials, Silesian University of Technology, Gliwice, Poland.

In the XPS experiments, a commercial XPS spectrometer (SPECS, Berlin, Germany) based on the UHV chamber was used, supported by an ion-sorption pump and equipped with an X-ray lamp (AlKα 1486.6 eV; XR-50 model, SPECS, Berlin, Germany). A concentric hemispherical analyzer (PHOIBOS-100 Model, SPECS, Berlin, Germany) combined with a steering and acquisition control unit as well as a commercial 3-axes sample manipulator with rotation (PREVAC, Rogow, Poland) completed the equipment. The XPS spectra registered in the various modes (survey, windows, and lines) were calibrated with respect to reference binding energies (BEs) using both an XPS Au4f peak at 84.5 eV of an Au foil also located on the sample holder as well as an XPS C1s peak at 284.5 eV of residual C contamination, normally present at the surface of investigated samples.

Then, in the temperature-programmed desorption (TPD) experiments, a commercial TDS spectrometer (PREVAC, Poland) with a UHV chamber was used, supported by a turbomolecular pumping system (Agilent, Santa Clara, CA, USA, TwisTorr 300 model) and equipped with the 3-axes sample manipulator with rotation, PREVAC, Rogow, Poland (with the resistive type heating unit), and residual gas analyzer (Stanford, Sunnyvale, CA, USA, RGA200 Model) for the detection of specific gases desorbed from the surface during the linear increase in the sample temperature, as controlled by a programmable power supply (HEAT3 PS model, PREVAC, Poland). The TDS spectra of selected gases including H_2_, H_2_O, O_2,_ and CO_2_ were recorded in the temperature range of 50–350 °C, corresponding to the typical ZnO gas sensor working conditions. Other experimental details of XPS and TDS studies have been described elsewhere [35,36].

## 3. Results and Discussion

**Figure 1:**
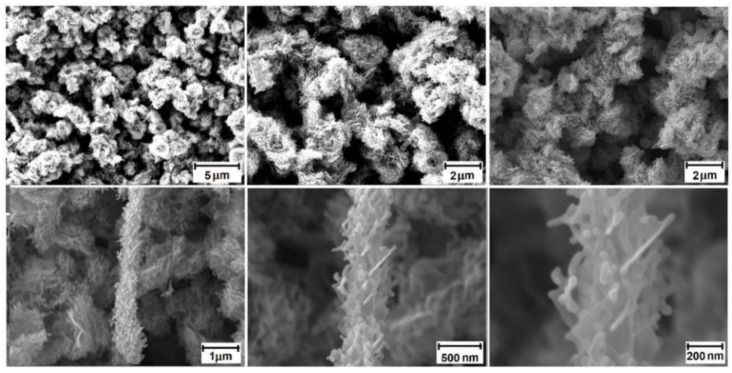
Set of SEM images of subsequent increased lateral resolution of flower-like ZnO nanostructures deposited on Si/SiO_2_ substrates.

First, a precise analysis of the surface morphology of the flower-like 3D ZnO nanostructures deposited on Si/SiO_2_ substrates was performed. Figure 1 shows the SEM images under increasing magnification and related lateral resolution, highlighting the surface morphological details.

From the SEM images shown in Figure 1, it can be observed that the flower-like ZnO nanostructures exhibit an evident non-planar and complex surface/subsurface morphology. They contain various, mostly isolated and irregular morphological low-dimensional forms, mainly rolled-up floss flowers with dimensions at the level of hundreds of nanometers, containing slightly joined short flosses of thicknesses at the level of several nanometers, together with local elongated complex stalks similar to Liatris flowers, containing joined short flosses in the form of nanodendrites of thicknesses at the level of several tenths of nanometer. The low-dimensional form of ZnO nanostructures may be responsible for their highly extended internal surface/subsurface, which plays a crucial role in the sensing mechanism of various gaseous compounds at the surface of metal oxide (MOX) nanomaterials. This is extremely important as the gas sensor effect appears mainly within the surface space-charge region of ZnO as a sensor material, related to the Debye length, which is at the level of several nanometers. After SEM investigations, we determined the surface chemistry of flower-like ZnO nanostructures using the XPS method, with a special emphasis on determining possible variations before and after the subsequent temperature-programmed desorption (TPD) procedure.

**Figure 2:**
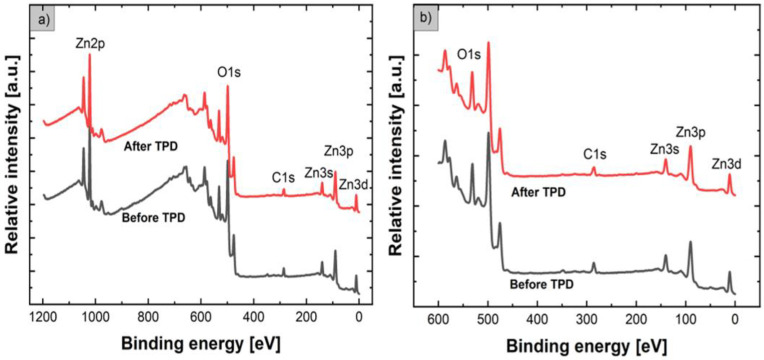
XPS survey spectra of flower-like ZnO nanostructures before and after the subsequent TPD process in the 1200 eV (**a**) and limited (600 eV, (**b**) binding energy ranges.

Figure 2 shows the XPS survey spectra for flower-like ZnO nanostructures before and after the TPD process in the typical full binding energy range of 0–1200 eV (Figure 2a), as well as in the limited binding energy range of 0–600 eV (Figure 2b).

From the XPS survey spectra in the full binding energy (BE) range (1200 eV) shown in Figure 2a, it is clearly visible that, apart from commonly observed high-energy-range Auger electron Zn LMM and O KLL peaks (not precisely labeled as not important), the contribution of ZnO results in core-level XPS lines such as Zn2p, O1s, Zn3s, Zn3p, and Zn3d, corresponding to the recognized basic elements. In addition, an evident contribution of C1s XPS lines at BE ~286.0 eV is also observed, confirming the existence of undesired carbon contamination at the surface, which can be treated as one of the most crucial points of our XPS studies. In general, according to the most commonly used analytical procedure, based on their respective atomic sensitivity factors [41,42], the relative concentrations of basic elements such as O, Zn, and C with respect to all atoms in the subsurface region were calculated based on Zn2p_3/2_, O1s, and C1s XPS core-level lines having the highest relative intensity (Figure 2b). However, because of the evident undesired high background in the XPS full binding energy (BE) range (1200 eV) survey spectra, including the contribution of Auger electron emission lines, we calculated the relative concentrations of the main elements of ZnO NFs primarily based on the relative intensity (height) of the O1s, C1s, and Zn3p core-level lines in a limited range (600 eV) (Figure 1), as corrected by the transmission function T(E) of the CHA PHOIBOS 100 energy analyzer. The data obtained are summarized in Table 1.

**Figure 3:**
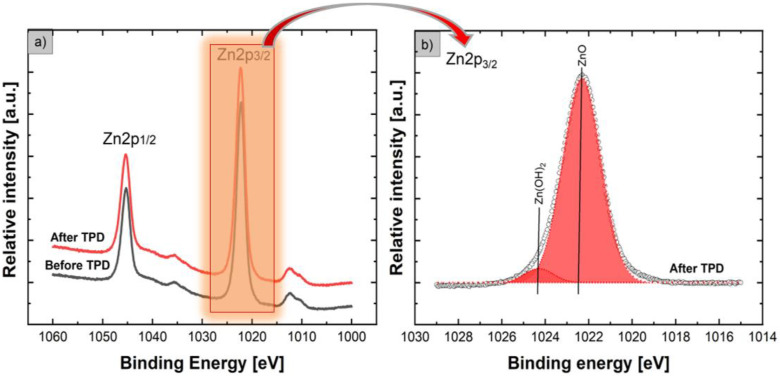
Evolution of core-level XPS Zn2p lines for flower-like ZnO nanostructures before and after the TPD process (**a**), together with the deconvoluted XPS Zn2p_3/2_ line after the TPD process (**b**), when using a Gaussian fitting procedure (dark circles (points)—experimental spectra; colored solid lines—respective fitted components).

The data summarized in Table 1 confirm the existence of evident non-stoichiometry at the surface of ZnO NFs. Before the TPD process, the relative concentrations of specific atoms such as Zn, O, and C with respect to all atoms in the subsurface region were ~0.49, ~0.27, and ~0.24, respectively, whereas, after the TPD process, these values changed to ~0.54, ~0.28, and ~0.18, respectively. This means that after the TPD process, only the relative concentration of O atoms with respect to all the above-mentioned atoms in the subsurface region of the ZnO NFs remained almost constant, whereas the relative concentration of Zn atoms with respect to all the main atoms in the subsurface region of the ZnO NFs slightly increased, which was directly related to the decrease in relative concentration of C atoms with respect to all atoms in the subsurface region, owing to the undesired contamination in the subsurface region of samples. This information confirms that ZnO nanoflowers are far from the natural stoichiometry. These effects were observed after the samples were exposed to air before gas sensor experiments. What is crucial is that these effects are critical for the potential use of our ZnO nanoflower devices as surface C contaminations can form a specific barrier for the gas interaction with expected surface sites, limiting the expected adsorption of various toxic and reactive compounds of interest. The evident non-stoichiometry at the surface of our ZnO NFs, combined with the existence of undesired surface C contamination, is probably related to the existence of and subsequent variation in the specific forms of surface bonding for the main elements in the subsurface region of the samples. Therefore, we performed a deeper analysis of the evolution of XPS Zn2p, O1s, and C1s peaks based on their deconvolution, to determine the main surface bonding before and after the TPD process. Figure 3 demonstrates the evolution of core-level XPS Zn2p lines for the flower-like ZnO nanostructures before and after the TPD process (Figure 3a), combined with the deconvoluted XPS 2p_3/2_ line after the TPD process (Figure 3b), when using the Gaussian fitting procedure.

**Figure 4:**
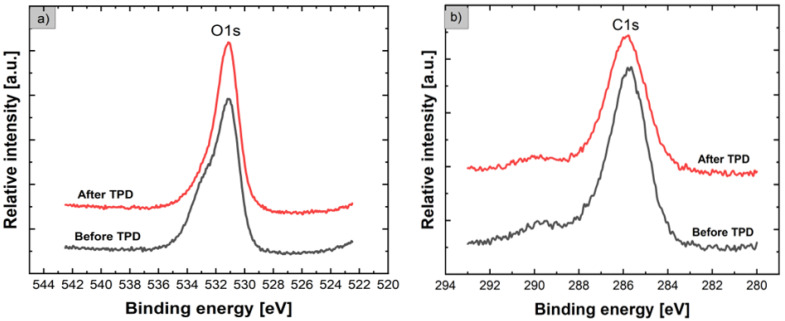
Evolution of core-level XPS O1s lines (**a**), as well as XPS C1s lines (**b**), for flower-like ZnO nanostructures before and after the subsequent TPD process.

From the XPS Zn2p lines shown in Figure 3a, it is clear that both samples (before and after TPD) contain two resolved XPS Zn2p_1/2_ and Zn2p_3/2_ peaks at binding energies of 1045 eV and 1022 eV, respectively. The existence of these two clearly resolved Zn2p_1/2_ and Zn2p_3/2_ peaks are related to Zn2p spin-orbit splitting, which was also observed in previously mentioned XPS studies [25,34]. The XPS Zn2p lines (before and after the TPD process) shown in Figure 3a are similar to one another and only slightly asymmetrical. Nevertheless, a precise deconvolution procedure was performed for the XPS Zn2p_3/2_ line showing a higher relative intensity after the TPD process. As can be observed from Figure 3b, the Zn2p_3/2_ line after deconvolution (with very high line fitting and with RMS equal to 0.995) can be divided into two peaks (components) located at binding energies of ~1022.3 eV and ~1024.3 eV. The first component at ~1022.3 eV confirms that for our ZnO NFs surfaces, the Zn element exists mainly in the form of Zn^2+^ ions in the ZnO lattice [31,34]. Then, the second component at ~1024.3 eV can be ascribed to the zinc hydroxide species. This second component was also observed for flower-like ZnO nanostructures by Bai et al. [31], as well as for ZnO nanoparticles as reported by Guo et al. [43] and for ZnO thin-films observed by Armelao et al. [44]. The existence of specific hydroxide species at the surface of flower-like ZnO nanostructures after the TPD process was also observed in the XPS O1s and XPS C1s spectral lines, as demonstrated and analyzed below. Figure 4 shows the evolution of core-level O1s and C1s lines for our flower-like ZnO nanostructures before and after the TPD process.

**Figure 5:**
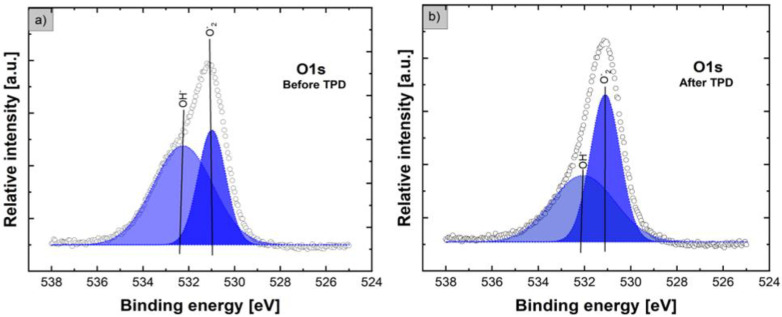
XPS O1s lines for flower-like ZnO nanostructures before (**a**) and after the subsequent TPD process (**b**), deconvoluted using the Gaussian fitting procedure (dark circles (points)—experimental spectra; colored solid lines—respective fitted components).

Concerning the O1s lines shown in Figure 4a, the relative intensities before and after the TPD process are similar, but their shape is evidently different. Before the TPD process, the core-level XPS O1s line exhibited an evident asymmetry, contrary to the sample after the TPD process, for which the asymmetry is greatly reduced. For the precise verification of the contribution of various forms of oxygen bonding at the surface of flower-like ZnO nanostructures, the deconvolution of XPS O1s lines before and after the TPD process was performed using the Gaussian fitting procedure. The results are shown in Figure 5.

**Figure 6:**
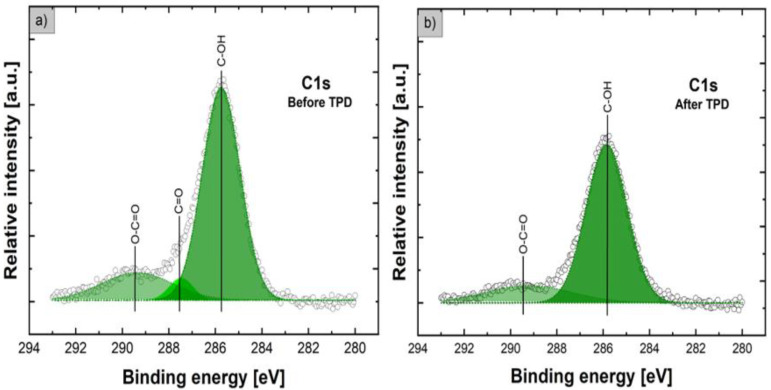
XPS C1s lines for flower-like ZnO nanostructures before (**a**) and after the subsequent TPD process (**b**), deconvoluted using the Gaussian fitting procedure (dark circles (points)—experimental spectra; colored solid lines—respective fitted components).

As can be noted in Figure 5a, before the TPD process, the core-level XPS O1s line, after the deconvolution procedure with a high line fitting parameter (RMS~0.98), exhibits two evident components observed at the binding energies (BEs) of 531.0 and 532.3 eV. The first, with an evidently higher relative intensity, located at 531.0 eV, can be assigned to the presence of partially reduced ZnO to ZnO_x_ and is related to the O_2_^−^ ions in oxygen-deficient regions inside the ZnO matrix. It is important that its binding energy is approximately 1 eV higher with respect to the commonly observed O_2_^−^ ions in the ZnO wurtzite structure of the hexagonal Zn ion array (~530 eV) [42]. A similar XPS O1s peak component located at ~531 eV was also recently observed for flower-like ZnO nanostructures by Bai et al. [31] and Chen et al. [33], as well as for various ZnO thin-films, as reported by Armelao et al. [44], Kaneva et al. [45], and Li et al. [46] and also for ZnO nanoparticles, as observed by Guo et al. [43]. The existence of oxygen-deficient regions originating from native defects such as oxygen vacancies and zinc interstitials at the surface of flower-like ZnO nanostructures is of great importance for their application as gas sensor materials. This is because these defects are mainly concentrated in the surface region and directly determine any variation in the electrical conductance, thus being directly responsible for the intrinsic sensing characteristics. The second component of the XPS O1s line located at 532.3 eV, evidently lower in relative intensity, is probably related to the existence of loosely bonded oxygen at the surface of our flower-like ZnO nanostructures in the form of specific species, mainly hydroxyl groups, as the direct consequence of H2O adsorption at their surfaces. A similar XPS O1s peak component attributed to the hydroxyl groups was also observed for flower-like ZnO nanostructures by Bai et al. [31] and Chen et al. [33], as well as for various ZnO thin-films by Armelao et al. [44], Hsieh et al. [47], Lupan et al. [48], and Stambolova et al. [49] and for various low-dimensional ZnO nanostructures by Kicir et al. [34], Guo et al. [43], Lee et al. [50], and Kim et al. [51]. As mentioned above, the existence of specific hydroxide species at the surface of the flower-like ZnO nanostructures after the TPD was also observed in the XPS C1s spectral line. As shown in Figure 4 right side, the relative intensities and shapes before and after the TPD process were similar. For the precise verification of the contribution of carbon bonding in various forms at the surface, deconvolution of XPS C1s lines before and after the TPD process was performed using the Gaussian fitting procedure. The results are shown in Figure 6.

As shown in Figure 6 left side, the core-level XPS C1s line after the deconvolution procedure, with a high line fitting parameter (RMS~0.98), contains three components clearly visible at binding energies (BEs) of 285.8, 287.5, and 289.3 eV. The first, with evidently the highest relative intensity, located at 285.8 eV, can only be assigned to C-O-type surface bonding, such as carbon hydroxyl C-OH groups, commonly observed at the surface of various forms of other oxides, as summarized in the well-known reference book [43]. Surprisingly, this XPS C1s peak component located at ~286 eV was not observed and analyzed in XPS studies on flower-like ZnO nanostructures recently performed by other groups [23,25,31,33,34]. The existence of surface carbon hydroxyl C-OH bonding is in good correlation with the observation of the existence of hydroxyl groups at the surface, as confirmed by the second component of the XPS O1s line located at 532.3 eV. The existence of this C-OH surface bonding is extremely important in chemical sensing applications because it can strongly affect active gas detection, especially at high levels of humidity. Two other components of the XPS C1s line after the deconvolution procedure (of evidently lower amplitude), located at the binding energies of ~287.5 and 289.3 eV, can be attributed to various forms of commonly observed carbon oxide bonding, such as C=O and O=C–O. Similar to the first XPS C1s component at 285.8 eV, they were not at all observed and analyzed in the XPS studies on similar ZnO nanostructures recently performed by other groups [23,25,31,33,34].

**Figure 7:**
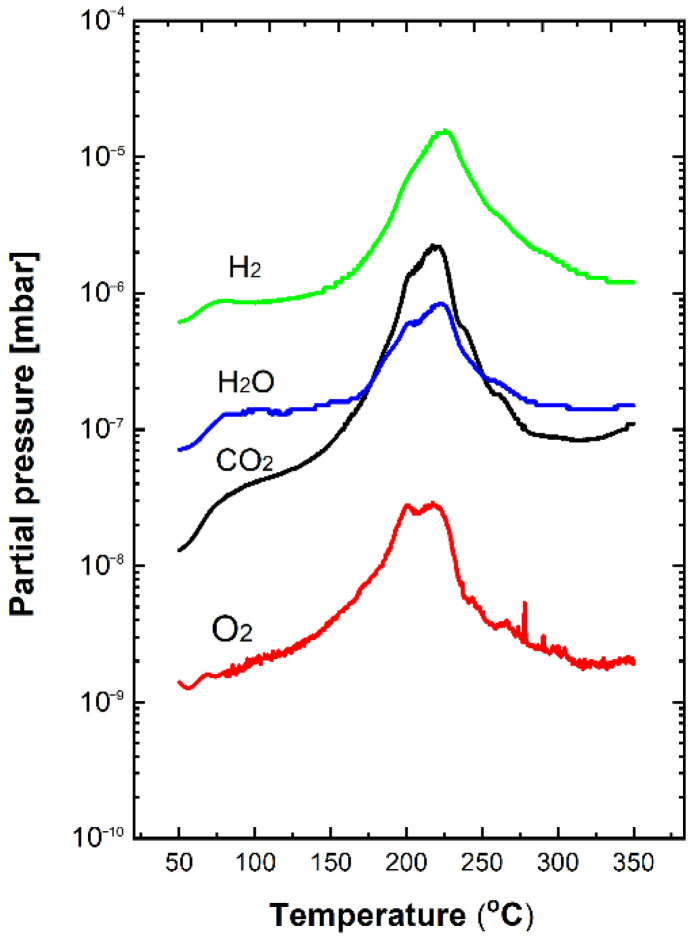
TDS spectra of the main residual gases desorbed from the flower-like ZnO nanostructures during the subsequent complementary TPD process.

Figure 6b shows the deconvolution of the core-level XPS C1s line after the TPD process, with a high line fitting parameter (RMS ~0.98), which contains only two components clearly visible at BEs of 285.8 and 289.3 eV. This means that after the TPD process, the C=O carbon oxide bonding was no longer observed. The information obtained from the XPS experiments is in quite good agreement with the information concerning the desorption effects of residual gases adsorbed at the surface of the flower-like ZnO nanostructures from the residual atmosphere, as obtained by the complementary TDS studies. The TDS spectra of the main residual gases desorbed from the surface in the temperature range 50–350 °C are displayed in Figure 7. For clarity, the obtained partial pressure values were additionally corrected by the ionization probability of the respective gases.

Molecular hydrogen (H_2_) exhibits the highest relative partial pressure, varying only one order of magnitude in the range 10^−6^–10^−5^ mbar. The desorption started at ~100 °C, and its maximum was observed at a temperature of ~220 °C. This is likely related to the fact that hydrogen, with the smallest molecules, can very easily penetrate the subsurface space of our flower-like ZnO nanostructures, mainly at room temperature (RT). To the best of our knowledge, this has never been reported in the literature. As mentioned above, in addition to hydrogen, carbon oxides (CO_x_) and water vapor (H_2_O) are the main residual gases that can always adsorb at the surface of ZnO nanostructures after exposure to the common air atmosphere. Therefore, in TDS studies, special attention was given to the analysis of the desorption effects of these two gases. As can be observed in the TDS spectra shown in Figure 7, carbon dioxide (CO_2_) was detected over a wide range (two orders of magnitude) of relative partial pressures of approximately 10^−8^–10^−6^ mbar, a wider range than that observed for molecular hydrogen (H_2_). It is also clearly visible that the desorption started from 50 °C, whereas the maximum relative partial pressure was observed at the temperature of ~220 °C, similar to the desorption of molecular hydrogen (H_2_).

Concerning water vapor (H_2_O) desorption, as can be seen in Figure 7, the relative partial pressure changed only by one order of magnitude in the range of approximately 10^−7^–10^−6^ mbar, starting below 100 °C, whereas the maximum relative partial pressures were observed at a temperature of ~220 °C, similar to the desorption of molecular hydrogen (H_2_) and carbon dioxide (CO_2_). This last observation correlates well with the XPS data analyzed above, given that the relative concentration of carbon with respect to all atoms in the subsurface region of flower-like ZnO nanostructures after the TDS process evidently decreased, as summarized in Table 1.

Finally, as can be seen in Figure 7, only a small amount of the molecular oxygen (O_2_) was desorbed from ZnO nanostructures with the lowest relative partial pressure with respect to molecular hydrogen (H_2_), carbon dioxide (CO_2_), and water vapor (H_2_O); this varied only in the range 10^−9^–10^−8^ mbar. It is also clear that the desorption started from 50 °C, whereas the maximum relative partial pressure was observed at the temperature of ~220 °C, similar to the desorption of other gases detected in the TDS experiments. This last observation is in agreement with the analyzed XPS data since the relative concentration of oxygen with respect to all other atoms in the subsurface region after the TDS process only decreased slightly, as summarized in Table 1.

Based on this analysis, it can be concluded that the residual gases from the air atmosphere detected in these studies can only be physically bonded to the surface of the flower-like ZnO nanostructures. This is important for the development of gas sensor devices since it confirms there is the possibility to remove, even partially, the undesired residual contamination from the surface of ZnO nanostructures during the outgassing process at a moderate temperature (around 200 °C). The removal of these contaminants (mainly carbon-based) may lead not only to better sensitivity of gas sensor devices based on this material but also to an improvement in their dynamic characteristics, such as response/recovery time(s), which could be shortened. These parameters are still the main limitations of metal oxide gas sensor devices, even when we apply low-dimensional nanostructures [6,7,8].

## 4. Conclusions

In this work, novel information regarding the local surface properties of flower-like ZnO nanostructures was described, correlating the surface morphology with the surface chemistry, based on comparative studies using the SEM, XPS, and TDS techniques. The investigated ZnO samples exhibited mostly isolated and irregular morphological low-dimensional forms, seen as rolled-up floss flowers containing slightly joined short flosses with a thickness of several nanometers, together with local elongated complex stalks similar to Liatris flowers, containing joined short flosses in the form of nanodendrites of thicknesses at the level of several tenths of a nanometer. Moreover, ZnO nanoflowers exhibit a slight surface nonstoichiometry mostly related to the existence of oxygen-deficient regions, combined with strong undesired C-surface contamination, as observed in our XPS studies. This last observation correlates well with the fact that this undesired surface contamination (including mainly C species and hydroxyl groups) is only slightly removed during the TDP process, causing an additional modification of the surface nonstoichiometry of our ZnO nanoflowers. The obtained results are of great importance for their potential application in novel gas sensor devices.

## Figures and Tables

**Table 1:** Relative concentrations of main elements at the surface of ZnO NFs before and after the temperature-programmed desorption (TPD) process.

ZnO NF	Relative Atomic Concentration		
	[Zn]/ ([Zn]+[O]+[C])	[O]/ ([Zn]+[O]+[C])	[C]/ ([Zn]+[O]+[C])
Before TPD	0.49	0.27	0.24
After TPD	0.54	0.28	0.18

## Data Availability

Not applicable.
